# Supplementary material for: Medical laboratory waste generation rate, management practices and associated factors in Addis Ababa, Ethiopia
Source: PLoS One. 2022 Apr 28;17(4):e0266888. doi: 10.1371/journal.pone.0266888 (PMC9049513; doi:10.1371/journal.pone.0266888)
Supplement: S1 File — (DOCX) [file pone.0266888.s001.docx]

**S1 file**

**S1 File. Questioner used for to assess the generation rate and management system of biomedical wastes and their associated factors in medical laboratories**

**Instruction: Please put √ or ×in the box provided.**

**Part I: Socio-demographic characteristics**

1. Sex: Male Female
2. Age: 21 – 30 years 31 – 40 years >40 years
3. Level of education

Diploma BSC MSC other

1. Work experience

< 1 year 1 – 5 years 5 – 10 years 10 – 15 years

15 – 20 years > 20 years

**Part II: Knowledge of biomedical waste management**

1. Do you know about biomedical waste management Policies and guidelines in Ethiopia?

Yes No

1. What are the common types of biomedical wastes? Especially in medical laboratories.

1. What are the basic components of biomedical waste management?

1. What are the most common infectious microorganisms transmitted during biomedical waste management?

1. What are the common health hazards associated with poor biomedical waste management?

1. What is the maximum time biomedical wastes could be kept in the facility premises?

< 12 hrs 12 -24 hrs 24 – 48 hrs > 48 hrs

1. Do you know the code of color for waste bins/buckets that suit a type of biomedical waste?

Yes No

If yes, mention them

1. What are the common modes of treatment of biomedical wastes before final disposal?

1. What are the appropriate methods for the final disposal of biomedical wastes?

1. When should a safety box be disposed of?

Full

¾ full

½ full

1. Have you received training in biomedical waste management?

Yes No

**Part III: Practice of biomedical waste management**

1. Does your health facility have standard operating procedures for biomedical waste management?

Yes No I don’t know

1. Are there separate containers for the collection of hazardous and non-hazardous biomedical wastes in your laboratory?

Yes No I don’t know

1. Are there color-coded bins/plastic buckets labeled with biohazard symbols for segregation and collection of biomedical waste in your laboratory?

Yes No I don’t know

1. Do containers of infectious waste have a biohazard symbol?

Yes No I don’t know

1. Are there safety boxes located in arm reach places for the collection of sharp wastes?

Yes No I don’t know

1. When do safety boxes get collected?

Full

¾ full

½ full

1. Are wastes holding plastic bag containers strong enough to resist puncture, leaking, and breaking under individual usage conditions?

Yes No I don’t know

1. Does the facility provide you with personal protective equipment like heavy-duty gloves, face masks, goggles, and duty shoes for waste handling?

Yes No I don’t know

1. What is the method of transport for biomedical wastes?

Use of bare hands

Use of wheelbarrows

Use of trolley

Other

1. What types of bins/ buckets are used for the transportation of biomedical wastes?

Open closed/container with lid other

1. Do infectious wastes be treated before disposing of?

Yes No I don’t know

1. Do reusable devices be cleaned and disinfected properly?

Yes No I don’t know

1. What are the common treatment methods of biomedical waste available in your health facility?

Incineration Autoclaving chemical disinfection

Other

1. How does liquid waste management in your laboratory?

Decontaminated first before dumping in running water

Dump into running water without decontamination

Other

1. What type of disposal method is used in the health facility?

Landfills Direct to municipal waste system Open burning pit

Other

1. Who is responsible for biomedical waste management in the laboratory?

Laboratory professionals

Administrative staffs

Sanitarians (Waste Handlers)

Others

1. Does the manager of this laboratory have a concern about biomedical waste management as their routine work?

Yes No I don’t know

1. Are you vaccinated for HBV and tetanus?

Yes No

1. Do you frequently wash your hand after handling biomedical wastes?

Yes No

1. Have you ever encountered physical injury while handling biomedical waste?

Yes No

If yes, list the type of injuries you faced

1. Is there a separate financial source for biomedical waste management?

Yes No I don’t know

If No, How does it get managed?

1. Does your facility legitimately follow the current guidelines of biomedical waste management in Ethiopia?

Yes No I don’t know

1. Do you think the current policy and guidelines regarding biomedical waste management in Ethiopia are enough for proper practice?

Yes No I don’t know

If no, what should be amended?

1. Do you separately dispose of personal protective equipment like a face mask, shield, gown, gloves, etc. in the infectious waste disposing bin?

Yes No

1. Does the infectious waste disposal of bins being used during the corona pandemic have a cover?

Yes No

# Annex II: Questioner for waste handlers

**Questionnaire for waste handlers to assess the generation rate and management system of biomedical wastes and its associated factors in medical laboratories**

**Instruction: Please put √ or ×in the box provided.**

**Part I: Socio-demographic characteristics**

1. Sex: Male Female
2. Age: < 21 years 21 – 30 years 31 – 40 years >40 years
3. Level of education

1 – 8 9 – 12 Diploma BSC other

1. Work experience

< 1 year 1 – 5 years 5 – 10 years 10 – 15 years

15 – 20 years > 20 years

**Part II: Biomedical waste management**

1. How is the availability of waste containers?

Available Not available

1. Do you collect biomedical wastes frequently?

Yes No I don’t know

If yes, how many times?

Every 12 hrs Every 24 hrs Every 48 hrs

Every week other

1. Are wastes holding plastic bag containers strong enough to resist puncture, leaking, and breaking under individual usage conditions?

Yes No I don’t know

1. Is there transporting equipment?

Yes No I don’t know

1. What is the quality of the transporting equipment?

Open With a closing lid

Plastic Impenetrable plastic

1. Does transporting equipment get disinfected or cleaned?

Yes No I don’t know

1. Is there enough and secured storage area?

Yes No I don’t know

1. How long the biomedical wastes get stored in the storage area before treatment or disposal?

< 12 hrs 12- 24 hrs 24 – 48 hrs > 48hrs

1. Is the waste storage room far from supply rooms and food preparation areas?

Yes No I don’t know

1. Do biomedical wastes get treated on-site or off-site?

On-site off-site I don’t know

1. What is the most common treatment method implemented in your facility?

Incineration Autoclaving chemical disinfection

Other

1. How do ashes or residues from incineration get managed?

1. Is there a permanent disposal site on the facility premises?

Yes No I don’t know

1. What is the most common disposal method implemented in your facility?

Open dumping Sanitary landfill Burial pit

Other

1. Does the facility provide you with personal protective equipment for biomedical waste handling?

Yes No I don’t know

1. Have you ever taken training in biomedical waste handling and management?

Yes No I don’t know

1. Have you ever encountered physical injury related to biomedical waste handling?

Yes No I don’t know

If yes, mention them

1. Do you frequently wash your hand after handling biomedical wastes?

Yes No

1. Do your facility/managers give a concern about biomedical waste handling?

Yes No I don’t know

1. Do you know about rules and regulations regarding biomedical waste management in our country?

Yes No I don’t know

1. Do you separately dispose of personal protective equipment like a face mask, shield, gown, gloves, etc. in the infectious waste disposing bin?

Yes No

# Annex III: Observational checklist

1. **Name of organization**
2. **Biomedical waste generation record**

| HC 1/ Hospital 1 | | Day 1 | Day 2 | Day 3 | Day 4 | Day 5 | Day 6 | Day 7 | Total | Remark |
| --- | --- | --- | --- | --- | --- | --- | --- | --- | --- | --- |
| Solid waste Kg/day | Hazardous |  |  |  |  |  |  |  |  |  |
|  | Sharp |  |  |  |  |  |  |  |  |  |
|  | Total |  |  |  |  |  |  |  |  |  |

# No of patients per day

# Available equipment

PPE

Safety box

Waste containers Reusable single-use

Labeled waste containers/color-coded bins

Transportation trolley or wheel burrow

1. **The practice of biomedical waste management**
2. Waste segregation practice

Color coding

Waste of corona virus pandemic ______________________

1. Waste collection practice

Time

Use of PPE during collection

Mechanism of collection

1. waste storage area

Is it far enough on the premises?

Is it open or secured?

1. Waste treatment

Incineration

Is it high temperature or low?

What is it made of?

Does it have an adequate air inlet and outlet?

Is it fenced?

Ash Remnant management

How are they managed?

Kept on an open field

Close dumping

Close dumping with cement

1. Waste disposal

Do biomedical wastes or ashes disposed of in a site away from any water source?

If landfills are used

Is there depth sufficient enough?

Is there a lime in the pit?

1. Liquid waste management

Do liquid wastes get disinfected before disposal?

Is there a septic tank
