# Supplementary material for: Medical laboratory waste generation rate, management practices and associated factors in Addis Ababa, Ethiopia
Source: PLoS One. 2022 Apr 28;17(4):e0266888. doi: 10.1371/journal.pone.0266888 (PMC9049513; doi:10.1371/journal.pone.0266888)
Supplement: Attachment — Submitted filename: Endris et al.docx [file pone.0266888.s002.docx]

Endris et al

The authors have submitted a manuscript that details a research project designed to evaluate the generation rate of biomedical waste, management practices and associated factors among public healthcare medical laboratories in Addis Ababa, Ethiopia. The topic is an important one across the globe and especially important in developing countries where resources are scarce. Public health officials, members of the health care delivery systems, administrators and the public have an interest in this evaluation. Unfortunately, the presentation does not do justice to the amount of work performed and the underlying value of the data generated. Although a considerable amount of useful data has been generated, the manuscript could be improved to provide more explicit information. Finally, the discussion section could be improved to provide the reader with the important take away messages generated by the research.

There are multiple components to the issue of biomedical waste from generation to transport to ultimate disposal. Efforts to limit production of biomedical waste, ensure safe and efficient transport and safe and permanent disposal without environmental impact are supported by a clear understanding of the current milieu. This research attempts to define the reality in hospitals and clinics in a large Ethiopian city. This type of evaluation would be especially useful to members of the organizations studied and especially laboratory managers.

The authors state their intent was to was conducted to “...assess the generation rate and management system of biomedical wastes of medical laboratories and its associated factors in Addis Ababa, Ethiopia which helps to identify factors affecting BMWM, initiate stakeholders to amend or develop more extensive policies and strategies and alarm laboratory managers to give attention to BMWM.

The authors may consider the following:

1. A depiction of the standard and acceptable handling of medical waste from generation to final disposal would be helpful.
   a. Is there a nationally recognized SOP?
   b. Do the organizations studied have clear SOP’s and if so how do they compare to national norms or regulations?
2. Compare the amount of medical waste generated in aggregate and by type of location (i.e. hospital or clinic).
   a. The manuscript provides aggregated data without providing a rationale for grouping what appears to be two distinctly different environments.
3. Compare the amount of waste generated by a standard comparative data set such as admissions, patients in hospital or patient visits to health centers. This would allow for a better understanding of the waste stream in hospitals as compared to health clinics. This will allow the identification of outlier institutions as compared to their peers.
   a. Table 6. Provides aggregated data but the methods by which the information was collected is not described in the manuscript.
4. The ability of both generators of biomedical waste (ie. Laboratory professionals) and disposal personnel to articulate the SOP’s coincident with their roles.
   a. There is evidence in the literature of dramatic differences based on roles and other factors
      i. (for example, see: Anand, P., Jain, R. and Dhyani, A., 2016. Knowledge, attitude and practice of biomedical waste management among health care personnel in a teaching institution in Haryana, India. *Int J Res Med Sci*, *4*(10), pp.4246-4250.)
   b. A rationale should be provided for aggregating the data
5. Compare and contrast the results of the self-administered questionnaires by occupation (waste handler/laboratory professional) and by site type.
   a. For example: professionals were queried on their understanding of eventual site of disposal (something that the waste handlers would be expected to know) as compared to the changing of sharps containers (something the laboratory personnel would be expected to know)
6. The data supports the contention that there are significant issues related to training and oversight. What is unclear is how institutional SOP’s differ from recognized national and international norms.
7. A good deal of work went into the generation of the data presented in the manuscript. Presumably such data would be informative to both the reader and to the administrators of the sites studied. While the data provided in Table 4. Is of interest, how is it of value to the readers or more importantly to the managers of the sites under study?

My specific comments:

Introduction:

- The introduction should provide a more meaningful context for the conduct of the study in Ethiopia. There is an inherent assumption that biomedical waste handling is not ideal locally, but the reader is provided with no supportive data. Presumably, since these data appear to have never been collected, a study of the performance of the sites is of value to those responsible for safe and efficient handling.
- Presumably, there are national or international standards that guide and inform biomedical waste handling that can act as a “gold standard” comparator to those identified at each healthcare location under study. This information is not explicitly provided.
- The authors provide a context for the study and the importance of the topic at hand. In doing so, they cite published works from India and South Africa and not Ethiopia. Readers would be well served by a clarification that the information provided is from other locations. Any relevant published works relative to the biomedical waste stream in general would be helpful to put these results in context. For example, what portion of the entire healthcare biomedical waste stream is generated from laboratories as compared to general care?
- The study was designed to assess the current volume of medical waste, management practices related to disposal and “initiate stakeholder to amend or develop more extensive policies and strategies and alarm laboratory managers to give attention” to the topic. The presentation of aggregated data may inhibit this goal and data should be presented so that any differences between the two type of sites would be evident.
- The study was conducted during the COVID-19 pandemic and is presumed to have produced in increased biomedical waste generation. While this may be intuitively obvious, the reader is not provided with any data supporting the contention. Data on waste generation per patient visit/stay pre and post COVID surge would be helpful to confirm this assumption

Materials and Methods

The study was conducted to:

- Daily measure the solid biomedical waste generated at each site
- Provide a self-administered questionnaire
- Determine actual practice using an observational checklist and interviews.
- Presumably local standard operating procedures for the study sites was the comparator. The SOP’s presumably meshed with Ethiopian national policy and guidelines. This is not made clear in the presentation.
- The nature of the questions posed in the self-administered questionnaire is loosely described. It is unclear if the waste handlers participated in the questionnaires as the document notes the emphasis on “knowledge of laboratory professionals regarding BMWM.”
- The authors note, “An observational checklist and interview were also used to

assess the actual practice of the BMWM system.” This is an important aspect of understanding the waste stream but there are no data provided on the observations.

- Table 6 in the results section provides data on waste generated based on the number of visits to the health centers. The methods section is silent on how these data were obtained. Similarly, no corresponding numbers are provided for the waste generated based on patient days in the hospital.

Operational definition of terms

- The authors refer to “three components of the biomedical waste system but do not describe what these are
- The WHO and others have standardized definitions of biomedical waste that should be referenced in this section
- The actual nature of the laboratories under study is not provided. Did they include anatomic pathology, chemistry, infectious disease, and other more specialized laboratories?
- Poor practice is defined in a somewhat confusing manner and does not appear to be used in the results or conclusions.

Results:

I find the results section nearly overwhelming with data and have a difficult time separating the wheat from the chaff. While the presentation of results in tabular format is acceptable, the authors may wish to consider highlighting the more important findings explicitly up front in this section and why the data presented are of importance.

Although there are perhaps other major takeaways, here are the ones that I find most interesting and disconcerting:

1. Half the laboratory professionals had received training on biomedical waste handling
2. Only 63% knew of policy and guidelines
3. 40% of the waste handlers indicated a separate process for handling biomedical waste
4. 67% of study participants indicated that their manager is concerned about biomedical waste handling
5. 18% reported encountering a sharp or needle stick injury.

Table 1. Sociodemographic data- it would be desirable to distinguish waste handlers from laboratory professionals in this regard.

Table 2. Presents results from laboratory professionals. Are there results associated with the waste handlers enrolled in the study? If so, it would be desirable to have as a comparison.

Table 3. Appears to be mislabeled as it represents only the studied institutions and not all in Addis Ababa

- The categorizations provided (total solid waste, infectious waste and sharp waste) were not previously described in the operational definition of terms
- The hospitals’ waste streams range from 6 to 36kg/day and the health centers from 3 to 28.4kg/day. Is there any identifiable reason for this range? Is it based on patient visits or specialty perhaps?

Table 4. Highlights the factors that appear to be related to knowledge. The authors may consider providing additional comments on their interpretation, (i.e., males with work experience of 1-5 years and previous training) can be used to improve compliance.

Discussion

My major concern with the discussion is that is simply provides data without a sophisticated assessment that I would have expected given the importance of the results and the sophistication of the authors.

- Were the study results provided back to the participating institutions? If so, was this done in aggregate or were results provided for each participating organization?
- The authors provide a data from an earlier study of biomedical waste generation from a hospital in Ethiopia noting similarities in biomedical waste volume. However, the comparator study included all biomedical waste generated not just that by the hospital laboratories.
- Once again the authors point to an apparent increase in biomedical waste generated as a result of the COVID-19 pandemic but no data are provided to support this notion
- The authors refer to the “three guidelines” regarding biomedical waste. The nature of these three guidelines is implied earlier in the manuscript but a more detailed explanation is warranted.
- The authors note that only 50.3% of participants received training in this study (a percentage that is well below what should be expected) but then take note that it is better than elsewhere in Ethiopia. The 50% training rate clearly is subpar and comparing it other locations does not address the underlying problem
- The authors note an 18.2% injury rate related to sharps seen during the study period. Were these injuries seen in waste handlers, laboratory professionals or both. Were appropriate precautions in place at the time that had they been followed would have prevented these events?

Lastly, what are the important takeaways identified in the exhaustive study? How can the situation be improved based on your findings?
