# Supplementary material for: Medical laboratory waste generation rate, management practices and associated factors in Addis Ababa, Ethiopia
Source: PLoS One. 2022 Apr 28;17(4):e0266888. doi: 10.1371/journal.pone.0266888 (PMC9049513; doi:10.1371/journal.pone.0266888)
Supplement: Attachment — Submitted filename: A rebuttal response letter to reviewers ABAY 9 Feb 22.docx [file pone.0266888.s003.docx]

**Date:** 9 February, 2022

**To: PLOS ONE Journal editorial office**

**Dear Editor:**

We are very much glad to write this response that our research output manuscript entitled **“Medical laboratory waste generation rate, management practices and associated factors in Addis Ababa, Ethiopia,** with a manuscript reference number **PONE-D-21-25475”** has been possible considered for publication in PLOS ONE. We are pleased to have an opportunity to make our manuscript revised and we have greatly appreciated for the reviewers’ high level comments, and suggestions were very helpful for the overall improvement of the manuscript. In revising the manuscript, we have carefully considered reviewers’ comments, academic editor and suggestions on our revised submission.

As instructed, we have attempted to concisely explain changes made in reaction to all comments and reply to each comment in point-by-point fashion as appended here

**Response to Academic Editor Comments/Journal requirements**

When submitting your revision, we need you to address these additional requirements.

**Comment # 1:** *Please ensure that your manuscript meets PLOS ONE's style requirements, including those for file naming. The PLOS ONE style templates can be found at*

*https://journals.plos.org/plosone/s/file?id=wjVg/PLOSOne_formatting_sample_main_body.pdf and https://journals.plos.org/plosone/s/file?id=ba62/PLOSOne_formatting_sample_title_authors_affiliations.pdf*

**Response#1.** The manuscript was prepared and revised as per the journal requirements.

**Comment #2**. *We suggest you thoroughly copyedit your manuscript for language usage, spelling, and grammar. If you do not know anyone who can help you do this, you may wish to consider employing a professional scientific editing service*

**Response#2**. As suggested, we have copyedited for language usage, spelling and grammar inviting an English writer colleague in our department. The changes were indicated by track changes and uploaded as ‘supportive information’*.*

**Comment # 3:** *Please include additional information regarding the survey or questionnaire used in the study and ensure that you have provided sufficient details that others could replicate the analyses. For instance, if you developed a questionnaire as part of this study and it is not under a copyright more restrictive than CC-BY, please include a copy, in both the original language and English, as Supporting Information.*

**Response** #**3**: As suggested, the questionnaire for the study was included as ‘additional information’ and cited in the manuscript. As S1.

Comment #4. Please improve statistical reporting and refer to p-values as "p<.001" instead of "p=.000". Our statistical reporting guidelines are available at: <https://journals.plos.org/plosone/s/submission-guidelines#loc-statistical-reporting>.

**Response #4**. As suggested, the manuscript was prepared following the statistical guidelines.

**Comment #5**. *Thank you for stating the following in the Acknowledgments Section of your manuscript:  We would like to acknowledge Addis Ababa University for giving us the opportunity and financing the research. Please note that funding information should not appear in the Acknowledgments section or other areas of your manuscript. We will only publish funding information present in the Funding Statement section of the online submission form.  Please remove any funding-related text from the manuscript and let us know how you would like to update your Funding Statement*.

**Response #5.** As suggested we have removed the funding information from the Acknowledgement section**, and modified as ‘…..**We would like to acknowledge Addis Ababa University for giving us the opportunity to conduct this study’ (line 455-456). We would like to amend the funding statement as: *This research was financed by Addis Ababa University. The funder had no role in study design, data collection, and analysis, the decision to publish, or preparation of the manuscript*’.

The authors may consider the following:

1. A depiction of the standard and acceptable handling of medical waste from generation to final disposal would be helpful.
   a. Is there a nationally recognized SOP?

**Response to reviewers:**

The country has three national health care waste management guidelines that detail SOPs for proper hazardous waste management. These are the Health Care Waste Management Directive developed by the Food, Medicine, and Healthcare Administration and Control Authority in 2005, the Healthcare Waste Management National Guidelines, Hygiene, and Environmental Health Development developed by the Federal Ministry of Health in 2008, and the Technical Guidelines on the Environmentally Sound Management of Biomedical and Healthcare Wastes developed by the Federal Environmental Protection Authority in 2004. These guidelines have been used and are still in use for more than ten years without being updated, and this study discovered that they lack the quality needed to implement a strong biomedical waste management practice.

b. Do the organizations studied have clear SOP’s and if so how do they compare to national norms or regulations?

**Response to reviewers:**

According to the study, 83.2 percent of study participants confirmed that their health facility has its own specific standard operating procedure developed in accordance with Ethiopia's national guidelines for health care waste management.

2. Compare the amount of medical waste generated in aggregate and by type of location (i.e. hospital or clinic).
   a. The manuscript provides aggregated data without providing a rationale for grouping what appears to be two distinctly different environments.

**Response to reviewers:**

Based on this constructive feedback, the biomedical waste generation rates of hospitals and health centers were calculated separately, yielding the following result. Hospitals in Addis Ababa generated 3.107 ± 2.016 kg/day of biomedical waste, of which 5.189 ± 3.807 kg/day was infectious waste and 1.026 ± 0.767 kg/day was sharp waste, whereas 20 sampled health centers in the city generated 2.331 ± 1.297 kg/day, consisting of 3.342 ± 1.762 Kg/day of infectious waste and 1.102 ± 0.843 kg/day of sharp waste.

Despite the fact that the study included a different number of hospitals and health centers, hospitals were found to generate more biomedical waste than health centers. This could be related to the disparity in the number of patients receiving laboratory services, with hospitals receiving 195.8 ± 50.621 patients per day while health centers provided the service to 86.55 ± 44.57 patients per day, as well as their breadth of service. The detail was depicted in table 3

3. Compare the amount of waste generated by a standard comparative data set such as admissions, patients in hospital or patient visits to health centers. This would allow for a better understanding of the waste stream in hospitals as compared to health clinics. This will allow the identification of outlier institutions as compared to their peers.
   a. Table 6. Provides aggregated data but the methods by which the information was collected is not described in the manuscript.

**Response to reviewers:**

The study only looked at the rate of biomedical waste generation in medical laboratories. Because medical laboratories in Addis Ababa do not provide a separate laboratory service for admitted and unadmitted patients, the variable called "number of patients" in this study only indicates the number of patients receiving the laboratory service per day.

The study compared the generation rate of biomedical wastes based on the number of patients and discovered that two hospitals (Zewditu Hospital and Triunesh Beijing Hospital) and five health centers (Kasanchis Health Center, Teklehaymanot Health Center, Mychew Health Center, Addis Ketema woreda 4 Health Center, and Entoto Number 2 Health Center) generated more biomedical waste than the other sample health institutions. (See the following table, table 4 of the revised manuscript.) Though it has been demonstrated that as the number of patients receiving laboratory services per day increases, so does the generation rate of biomedical waste, with a positive estimate of t – value of 3.032 and p – value of.006.(table 7).

| **Variable** | **Number of patients** | | **Name of the organization** | **Value** |
| --- | --- | --- | --- | --- |
| **Solid waste generation Total Kg/day** | 60 | Highest | Kasanchis HC | 7.8 |
|  |  | Lowest | Hiwot Amba HC | 6.2 |
|  | 70 | Highest | Teklehymanot HC | 16.70 |
|  |  | Lowest | Addis Ketema W7 HC | 7 |
|  | 80 | Highest | Mychew HC | 28.40 |
|  |  |  | Addis Ketema W4 HC | 19.60 |
|  |  | Lowest | Ferency W1 HC | 3 |
|  |  |  | Arada HC | 7.5 |
|  | 120 | Highest | Entoto No.2 HC | 29 |
|  |  | Lowest | Nifas selk W02 HC | 8.5 |
|  | 200 | Highest | Zewdiut Hospital | 36 |
|  |  |  | Triunesh Beijing Hospital | 19.10 |
|  |  | Lowest | Goro HC | 11 |
|  |  |  | Bisrate Gebirel HC | 14.90 |

4. The ability of both generators of biomedical waste (i.e. Laboratory professionals) and disposal personnel to articulate the SOP’s coincident with their roles.
   a. There is evidence in the literature of dramatic differences based on roles and other factors
      i. (For example, see: Anand, P., Jain, R. and Dhyani, A., 2016. Knowledge, attitude and practice of biomedical waste management among health care personnel in a teaching institution in Haryana, India. *Int J Res Med Sci*, *4*(10), pp.4246-4250.)
   b. A rationale should be provided for aggregating the data

**Response to reviewers**:

The study employed a separate assessment tool of biomedical waste management practice for laboratory professionals, which was narrated in response to the questioner.

For example, the majority of 228 (63%) of laboratory professionals and 52 (81.3%) of waste handlers in this study were aware of Ethiopia's biomedical waste management policy and guidelines.

Laboratory professionals were quizzed on their knowledge of all aspects of biomedical waste management, whereas waste handlers were quizzed on the aspects for which they are responsible.

Furthermore, the study aimed to assess the practice of biomedical waste management in public health care facilities using unbiased sources of information such as laboratory professionals and waste handlers, as well as observation, rather than an interview with laboratory and health care facility managers and responsible persons. Despite the fact that the study discovered that knowledge about biomedical waste management is associated with proper practice of its management in health care facilities, and knowledge questions were included in the questioner, assessing and comparing personal knowledge, attitude, and practice was not the study's goal.

Because personal knowledge was discovered to be one of the factors associated with biomedical waste management practice, we included it in the recommendation section for future research on personal knowledge, attitude, and practice on biomedical waste management.

5. Compare and contrast the results of the self-administered questionnaires by occupation (waste handler/laboratory professional) and by site type.
   a. For example: professionals were queried on their understanding of eventual site of disposal (something that the waste handlers would be expected to know) as compared to the changing of sharps containers (something the laboratory personnel would be expected to know)

**Response to reviewers:**

All biomedical waste management components were evaluated by both laboratory professionals and waste handlers in order to obtain more precise data that was confirmed and crosschecked on both sides. Waste handlers were asked about the components they were most likely involved with, such as waste collection, transportation, storage, treatment, and disposal. The findings were confirmed by observation of the actual sites, in addition to the data obtained from questioners.

The results obtained from laboratory professionals and waste handlers were found to be more or less similar and to confirm one another. For example, in waste transportation practice, 144 (48.3 %) and 24 (8.1 %) of laboratory professionals indicated that the methods used for transportation of biomedical wastes are holding the waste-collecting bags with bare hands and by the waste container itself, respectively. 26 (40.6 %) of waste handlers also demonstrated that there is no separate biomedical waste transportation tool in the facility.

6. The data supports the contention that there are significant issues related to training and oversight. What is unclear is how institutional SOP’s differ from recognized national and international norms.

**Response to reviewers:**

The study only used a questioner to assess the presence of separate SOP for biomedical waste management in sampled health care facilities. However, it did not attempt to evaluate the content and differences between each SOP and the national guideline. This could be interpreted as a limitation, and we recommend that it be considered in future studies.

7. A good deal of work went into the generation of the data presented in the manuscript. Presumably such data would be informative to both the reader and to the administrators of the sites studied. While the data provided in Table 4. Is of interest, how is it of value to the readers or more importantly to the managers of the sites under study?

**Response to reviewers:**

For seven days, researchers measured the quantity of biomedical waste generated in medical laboratories per kilogram every day. Table 4 summarizes the total amount of biomedical waste generated and the number of patients receiving laboratory services in each health care facility studied, as well as the mean and standard deviation for each. As a result, it provides both readers and administrators with an overall picture of the rate of biomedical waste generation in the city and in each specific health care facility. It will also make it easier to correlate the pace of generation with the number of patients who receive medical laboratory services. This also distinguishes which health-care facility has to improve its biomedical waste management in order to reduce the rate.

**My specific comments:**

**Introduction:**

- The introduction should provide a more meaningful context for the conduct of the study in Ethiopia. There is an inherent assumption that biomedical waste handling is not ideal locally, but the reader is provided with no supportive data. Presumably, since these data appear to have never been collected, a study of the performance of the sites is of value to those responsible for safe and efficient handling.
- Presumably, there are national or international standards that guide and inform biomedical waste handling that can act as a “gold standard” comparator to those identified at each healthcare location under study. This information is not explicitly provided.
- The authors provide a context for the study and the importance of the topic at hand. In doing so, they cite published works from India and South Africa and not Ethiopia. Readers would be well served by a clarification that the information provided is from other locations. Any relevant published works relative to the biomedical waste stream in general would be helpful to put these results in context. For example, what portion of the entire healthcare biomedical waste stream is generated from laboratories as compared to general care?
- The study was designed to assess the current volume of medical waste, management practices related to disposal and “initiate stakeholder to amend or develop more extensive policies and strategies and alarm laboratory managers to give attention” to the topic. The presentation of aggregated data may inhibit this goal and data should be presented so that any differences between the two type of sites would be evident.
- The study was conducted during the COVID-19 pandemic and is presumed to have produced in increased biomedical waste generation. While this may be intuitively obvious, the reader is not provided with any data supporting the contention. Data on waste generation per patient visit/stay pre and post COVID surge would be helpful to confirm this assumption

**Response to reviewers:**

Based on this constructive feedback, in the revised manuscript the authors incorporated supporting data ranging from global data to systematic reviews conducted in Ethiopia regarding health care waste management practices in public health care facilities, providing a more meaningful context and justifying the need for this study. The study's aggregated data on the rate of biomedical waste generation is likewise scattered to give a clear view of the two diverse study settings.

We were unable to compare the pre and post COVID generation rates since there is no recorded data on past generation rates of biological waste in sampled medical laboratories and information years ago was obtained to indicate the entire health care facility.

Misinterpretations of information have now been corrected, as the COVID-19 pandemic has made professionals aware of the importance of segregating waste in waste collection bins with covers related to the pandemic.

**Materials and Methods**

The study was conducted to:

- Daily measure the solid biomedical waste generated at each site
- Provide a self-administered questionnaire
- Determine actual practice using an observational checklist and interviews.
- Presumably local standard operating procedures for the study sites was the comparator. The SOP’s presumably meshed with Ethiopian national policy and guidelines. This is not made clear in the presentation.
- The nature of the questions posed in the self-administered questionnaire is loosely described. It is unclear if the waste handlers participated in the questionnaires as the document notes the emphasis on “knowledge of laboratory professionals regarding BMWM.”
- The authors note, “An observational checklist and interview were also used to assess the actual practice of the BMWM system.” This is an important aspect of understanding the waste stream but there are no data provided on the observations.
- Table 6 in the results section provides data on wastes generated based on the number of visits to the health centers. The methods section is silent on how these data were obtained. Similarly, no corresponding numbers are provided for the waste generated based on patient days in the hospital.

**Response to reviewers:**

Despite the fact that most medical laboratories and public health care facilities have developed their own standard operating procedures for biomedical waste management, the study defined and contrasted the practice of biomedical waste management using national regulations and standards. The results of medical laboratory observation are now included in the manuscript, as well as a data collection tool for waste handlers.

**Operational definition of terms**

- The authors refer to “three components of the biomedical waste system but do not describe what these are
- The WHO and others have standardized definitions of biomedical waste that should be referenced in this section
- The actual nature of the laboratories under study is not provided. Did they include anatomic pathology, chemistry, infectious disease, and other more specialized laboratories?
- Poor practice is defined in a somewhat confusing manner and does not appear to be used in the results or conclusions.

**Response to reviewers:**

Under the definition of the biomedical waste management system, the operational definition part specified the seven components of biomedical waste management. According to this definition, health care facilities that do more than three components properly are considered to be practicing well, whereas those that accomplish less than three components are considered to be practicing poorly.

Sampled medical laboratories in health care facilities, though in various settings, include hematology, chemistry, microbiology, parasitology, and serology departments, which are a common structure for all health care facilities in Addis Ababa, Ethiopia.

**Results:**

I find the results section nearly overwhelming with data and have a difficult time separating the wheat from the chaff. While the presentation of results in tabular format is acceptable, the authors may wish to consider highlighting the more important findings explicitly up front in this section and why the data presented are of importance.

Although there are perhaps other major takeaways, here are the ones that I find most interesting and disconcerting:

1. Half the laboratory professionals had received training on biomedical waste handling
2. Only 63% knew of policy and guidelines
3. 40% of the waste handlers indicated a separate process for handling biomedical waste
4. 67% of study participants indicated that their manager is concerned about biomedical waste handling
5. 18% reported encountering a sharp or needle stick injury.

Table 1. Sociodemographic data- it would be desirable to distinguish waste handlers from laboratory professionals in this regard.

Table 2. Presents results from laboratory professionals. Are there results associated with the waste handlers enrolled in the study? If so, it would be desirable to have as a comparison.

Table 3. Appears to be mislabeled as it represents only the studied institutions and not all in Addis Ababa

- The categorizations provided (total solid waste, infectious waste and sharp waste) were not previously described in the operational definition of terms
- The hospitals’ waste streams range from 6 to 36kg/day and the health centers from 3 to 28.4kg/day. Is there any identifiable reason for this range? Is it based on patient visits or specialty perhaps?

Table 4. Highlights the factors that appear to be related to knowledge. The authors may consider providing additional comments on their interpretation, (i.e., males with work experience of 1-5 years and previous training) can be used to improve compliance.

**Response to reviewers**:

For a better understanding of readers, the result session is rewritten based on the coherence of the questioner, tables are reformatted, and requested operational definitions are added.

As described in the methodology section, waste handlers were quizzed with other questioners focused on the practice of biomedical waste management in the facility, and the data in table 2.shows only laboratory professionals' knowledge assessment.

**Discussion**

My major concern with the discussion is that is simply provides data without a sophisticated assessment that I would have expected given the importance of the results and the sophistication of the authors.

- Were the study results provided back to the participating institutions? If so, was this done in aggregate or were results provided for each participating organization?
- The authors provide a data from an earlier study of biomedical waste generation from a hospital in Ethiopia noting similarities in biomedical waste volume. However, the comparator study included all biomedical waste generated not just that by the hospital laboratories.
- Once again the authors point to an apparent increase in biomedical waste generated as a result of the COVID-19 pandemic but no data are provided to support this notion
- The authors refer to the “three guidelines” regarding biomedical waste. The nature of these three guidelines is implied earlier in the manuscript but a more detailed explanation is warranted.
- The authors note that only 50.3% of participants received training in this study (a percentage that is well below what should be expected) but then take note that it is better than elsewhere in Ethiopia. The 50% training rate clearly is subpar and comparing it other locations does not address the underlying problem
- The authors note an 18.2% injury rate related to sharps seen during the study period. Were these injuries seen in waste handlers, laboratory professionals or both. Were appropriate precautions in place at the time that had they been followed would have prevented these events?

Lastly, what are the important takeaways identified in the exhaustive study? How can the situation be improved based on your findings?

**Response to reviewers**:

The study's findings have not yet been distributed to the participating institutions. The discussion section compares the findings of this study with available data on biomedical waste management, where the only available data is from studies conducted in the entire health care facility, not just in medical laboratories.

This section has also been modified and revised in the revised manuscript in accordance with the reviewers' suggestions.
